# Notch3 regulates Mybl2 via HeyL to limit proliferation and tumor initiation in breast cancer

**DOI:** 10.1038/s41419-023-05674-7

**Published:** 2023-02-28

**Authors:** Sonia Brahim, Ana-Maria Negulescu, Clara Geneste, Thomas Schott, Shuheng Lin, Louis-Oscar Morel, Nicolas Rama, Nicolas Gadot, Isabelle Treilleux, Patrick Mehlen, Olivier Meurette

**Affiliations:** 1 Apoptosis, Cancer and Development Laboratory—Equipe labellisée ‘La Ligue’, LabEx DEVweCAN, Centre de Recherche en Cancérologie de Lyon, INSERM U1052-CNRS UMR5286, Université de Lyon, Centre Léon Bérard, 69008 Lyon, France; 2 Laboratory of Stem Cells and Cancer, Université Libre de Bruxelles, Brussels, Belgium; 3 Centre Léon Bérard, Pathology Department, 69008 Lyon, France; 4 Centre Léon Bérard, Department of Translational Research and Innovation, 69008 Lyon, France

**Keywords:** Breast cancer, Cell signalling

## Abstract

Notch signaling is a conserved signaling pathway that participates in many aspects of mammary gland development and homeostasis, and has extensively been associated with breast tumorigenesis. Here, to unravel the as yet debated role of Notch3 in breast cancer development, we investigated its expression in human breast cancer samples and effects of its loss in mice. Notch3 expression was very weak in breast cancer cells and was associated with good patient prognosis. Interestingly, its expression was very strong in stromal cells of these patients, though this had no prognostic value. Mechanistically, we demonstrated that Notch3 prevents tumor initiation via HeyL-mediated inhibition of Mybl2, an important regulator of cell cycle. In the mammary glands of Notch3-deficient mice, we observed accelerated tumor initiation and proliferation in a MMTV-Neu model. Notch3-null tumors were enriched in Mybl2 mRNA signature and protein expression. Hence, our study reinforces the anti-tumoral role of Notch3 in breast tumorigenesis.

## Introduction

Notch signaling, through one of four family members (Notch 1, 2, 3, 4), is aberrantly regulated in many different cancers [1] and plays pleiotropic roles in the tumor microenvironment [2]. Upon interaction with a ligand of the Delta/Serrate/LAG (DSL) family, the Notch intracellular domain (NICD) is released and translocates to the nucleus where it regulates transcription [3]. In breast cancers, activation of Notch signaling is largely associated with oncogenic properties [4–6], and Notch1 signaling induced resistance to chemotherapy in vitro [7]. Nevertheless, the role of the less extensively studied Notch3 in mammary gland tumorigenesis remains debated, as it has been described as on oncogene [8–12] and as a tumor suppressor [13–15]. Of note, most of these studies were performed in vitro on cell lines and no in vivo model of Notch3 loss in a context of mammary gland tumor development has so far been conducted to unravel its precise role. Interestingly, in mouse mammary glands, Notch3 is expressed by luminal progenitor cells, described as the cell of origin of luminal, HER2 and basal breast cancers [16–18], and inhibits their proliferation [19], suggesting that it may have a tumor suppressive activity.

Here, we initially characterized Notch3 expression in human breast cancers using Tumor Micro Array (TMA) and observed a decrease in Notch3 expression in epithelial cancer cells associated with decreased patient survival. In parallel, we observed that mice developed mammary gland tumors more rapidly and had a shorter tumor-free survival after Notch 3 inactivation than Notch3-positive tumors. Loss of Notch3 was associated with a proliferative signature, including increased Mybl2, which was confirmed by in silico analysis in human tumors. Mechanistically, in vitro expression of Notch3 in organoïds induced a HeyL-dependent reduction of Mybl2 leading to reduced proliferation. In human tumors, HeyL was also associated with a decrease in the Mybl2 signature. We therefore conclude that a Notch3-HeyL-Mybl2 axis limits proliferation in breast cancers.

## Results

### Notch3 expression is decreased in breast cancer patients

**Fig. 1:**
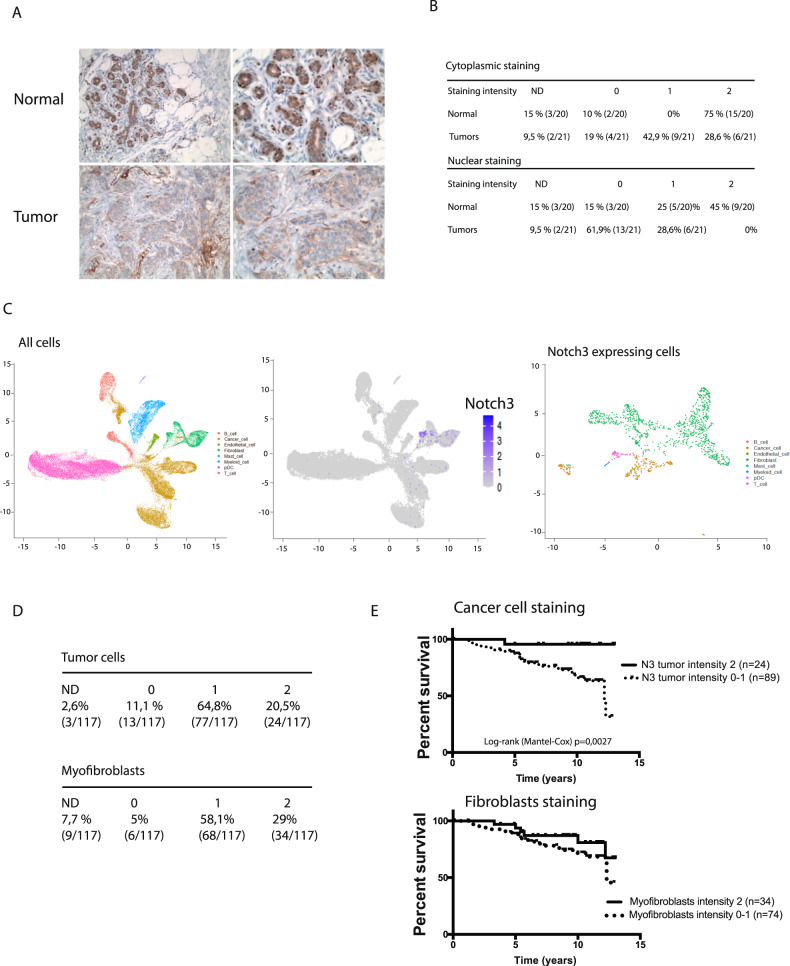
Notch3 is lost and of good prognosis in breast cancers. **A** Immunohistochemistry of Notch3 expression (Notch3 D11B8 Cell Signaling antibody) in normal or tumor tissue. **B** Quantification of Notch3 staining in cancer cells of 21 infiltrating Breast cancer and adjacent histological normal tissu. **C** Analysis of Notch3 expression in breast cancer in single-cell data from Bassez et al. **D** Repartition of Notch3 staining intensity in cancer cells and myofobroblasts in a TMA of 117 breast cancer patients. **E** Kaplan–Meier plot of 117 patient’s survival depending on Notch3 expression localization: cancer cells or fibroblasts (TMA cohort).

To clarify the role of Notch3 in breast cancers, we first assessed Notch3 protein expression by immunohistochemistry in 21 paired infiltrating adenocarcinoma/adjacent healthy tissue (Fig. 1A). As expected from previous studies [12], Notch3 protein staining in the breast cancer epithelial compartment was weak (only 28.6% of tumor tissues had a strong staining intensity compared to 75% (15/20) of the normal tissues) (Fig. 1B), whereas it was strong in stromal cells and in the vascular system (Fig. 1A). Based on previously published datasets on Notch3 mRNA expression in breast cancer and melanoma [14], we confirmed that Notch3 mRNA expression was significantly decreased in all breast cancer subtypes (Supplementary Fig. S1A). However, this dataset contains a small number of normal unmatched tissues and we observed no decrease in Notch3 mRNA expression in the TCGA-BRCA dataset using matched tumor/normal samples (Supplementary Fig. 1B). Of note, the use of bulk RNA sequencing data does not reveal the cell-intrinsic role of Notch3 in tumor cells. Next, we therefore used published data of single-cell sequencing [20] to assess the expression of Notch3 in the different compartments of tumors. Notch3 expression was present in a small proportion of cells and enriched in the pericyte/fibroblast cell population, particularly fibroblasts expressing PDGFRB and ACTA2, confirming our immunohistochemistry observation (Fig. 1C). Among the cancer cells expressing Notch3, two distinct clusters of epithelial cancer cells were visible, but most of the cells were fibroblasts in every subtype of breast cancer (Supplementary Fig. 1C). To verify the impact of Notch3 expression in cancer versus stromal cells (fibroblasts), we analyzed a TMA containing 117 samples of breast cancer patients. We analyzed Notch3 expression in myofibroblast and in cancer cells (Supplementary Fig. 2). Regarding the stromal compartment, Notch3 was expressed in the vessels in all patients and in myofibroblasts in 87.1% of the patients with a strong staining in 29% of these (Fig. 1D). Regarding the expression in tumor cells, the staining was strong (intensity = 2) in 20% of the patients, corroborating our previous results and those presented in other studies (Fig. 1B, C) [12]. Finally, we assessed the prognostic value of Notch3 expression in these two compartments. In both cases, we compared strong expression (staining intensity = 2) with moderate or no expression (staining intensity = 0 or 1). We observed a significant increase in patient survival when Notch3 expression was strong in tumor cells (Fig. 1E), whereas stromal expression had no impact on survival (Fig. 1E), arguing in favor of a tumor cell-intrinsic role for Notch3 in breast cancers.

### Inhibition of Notch3 expression is regulated by the methylation of its promoter

**Fig. 2:**
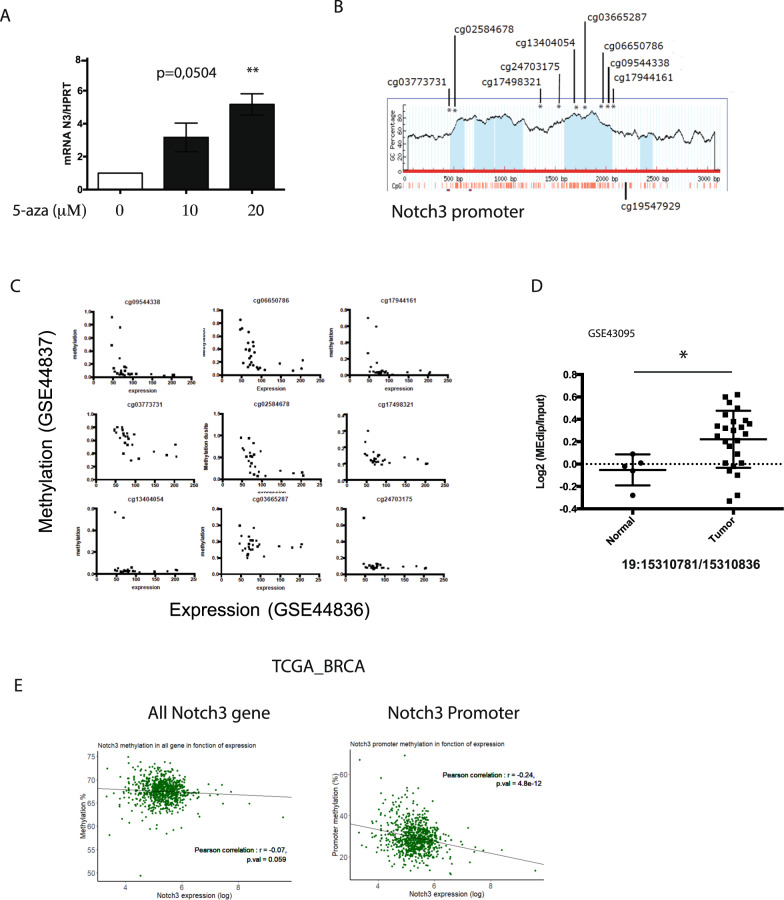
Notch3 promoter methylation regulates Notch3 expression in breast cancer. **A** mRNA Notch3 expression in MDA-MB231 cells treated with 0 (control), 10 or 20 μM 5-aza for 24 h assessed by RT-qPCR. **B** Notch3 promoter CpG island map with probes from the 450 K chip. **C** Correlation between methylation and expression of Notch3 for 9 CpG probes in 26 breast cancer cell lines (GSE44836 and GSE44837 datasets). **D** Methylation of Notch3 promoter region in 5 normal breast samples and 20 breast cancer samples (GSE43095 dataset). Student *t*-test was used. **E** Correlation between the mean of CpG methylation in all Notch3 gene (left) and in first CpG island situated in the promoter of Notch3 (right) and Notch3 expression (TCGA data).

Next, we sought the mechanism(s) by which Notch3 expression was reduced in breast cancer. As methylation of the Notch3 gene promoter has been described in breast cancer cell lines [21], we sought to confirm these data and expand to large published datasets. We thus treated cells of the human adenocarcinoma cell line, MDA-MB-231, that express very low amounts of Notch3, with 5-azacitidine (5-aza) a well-known DNA demethylation agent. As observed by [21] we observed re-expression of Notch3 mRNA, suggesting that DNA methylation could repress Notch3 expression (Fig. 2A). We then identified CpG probes in a CpG island flanking the TSS (transcription start site) of Notch3 (Fig. 2B) and looked for methylation in breast cancer cell lines. We observed a negative correlation between Notch3 methylation and Notch3 expression using the GSE44837 and GSE44826 dataset containing data on mRNA expression and methylation in the same breast cancer cell lines (Fig. 2C). We confirmed by pyrosequencing, the differential methylation of MDA-MB-231 and MCF7 cells, focusing on CpG probes from the previously mentioned datasets (Supplementary Fig. S1D). In the patient breast cancer dataset (GSE43095), we observed an increased methylation of the Notch3 promoter in tumor samples compared to normal tissue (Fig. 2D). TCGA breast cancer dataset (BRCA) analysis confirmed a weak but significant negative correlation between Notch3 expression and Notch3 methylation in the first CpG island that includes the promoter, the first exon and part on the first intron (Fig. 2E and Supplementary Table S1). We then specifically focused on the CpG probes located in the first CpG island of Notch3 and observed a significant negative correlation between Notch3 methylation and expression for all CpG probes within the first CpG island (Supplementary Table S1). In addition, the negative correlation between methylation and expression was also valid, albeit to a lesser extent, for the second CpG Island localized in the second intron of Notch3.

Using TCGA paired samples (normal and tumor), we then looked for methylation events in patients with a decreased Notch3 expression in the tumor sample (T/N < 1) and patients with no decreased Notch3 expression (T/N > 1). Methylation of different probes was higher in patient’s tumors displaying lower Notch3 expression (Supplementary Fig. 1E). Furthermore, in the methylation clusters published by the TCGA consortium [22], we observed that Notch3 expression was higher in the cluster 5, in which methylation is low, further reinforcing the correlation between methylation and Notch3 expression (Supplementary Fig. 1F). These data confirmed that methylation in the promoter region of Notch3 could have a silencing effect on transcription and contribute to decreased Notch3 expression in breast cancers.

### Notch3 limits tumor initiation in mouse mammary glands

**Fig. 3:**
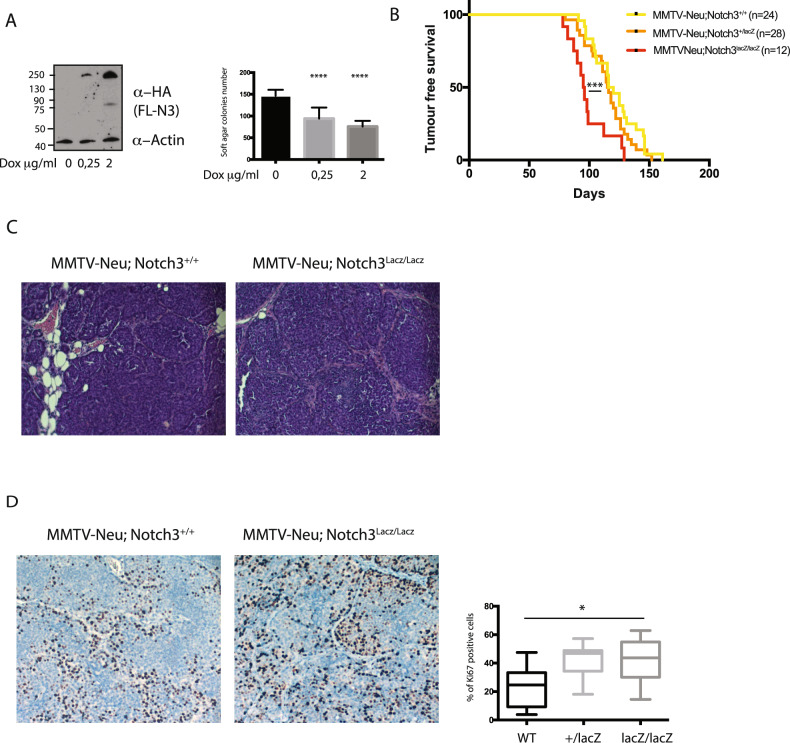
Notch3 loss accelerates proliferation and tumor ontake in a MMTV-Neu mammary gland tumor model. **A** Western blot of Notch3 expression following treatment with Doxycycline of MDA-MB231 cells stably expressing inducible Notch3 plasmid and quantification of soft-agar colony. 0.25 μg/mL or 2 μg/mL of Doxycyclin (DOX) was added in the cell medium every 3 days. **B** Kaplan–Meier plot of tumor-free survival of MMTV-Neu; Notch3^+/+^, MMTV-NEU; Notch3^+/lacZ^ and MMTV-NEU; Notch3^lacZ/lacZ^ mice. **C** Hematoxylin staining of tumors dissected from MMTV-NEU; Notch3lacZ/lacZ or MMTV-Neu/Notch3^+/+^ mice. **D** Immunohistochemistry using Ki67 antibody of tumors dissected from MMTV-NEU; Notch3^lacZ/lacZ^ or MMTV-Neu; Notch3^+/+^. Right panel shows quantification using HALO software.

Having correlated Notch3 expression with good breast cancer patient prognosis, we wondered whether Notch3 signaling could limit cancer cell transformation. We thus conducted soft-agar assays to measure colony formation of MDA-MB231 cells engineered to express Notch3 upon doxycycline treatment. Forced Notch3 expression induced a dose-dependent reduction of colony formation (Fig. 3A), demonstrating a cell autonomous effect of Notch3 in limiting breast cancer progression, supporting the good prognostic impact of Notch3 expression observed in cancer cells (Fig. 1E).

The effect of Notch3 loss was then investigated in vivo in a model of mammary gland tumors. To do so, we crossed the MMTV-Neu mice [23], with Notch3 loss-of-function mice [24]. We monitored tumor initiation every 2 days from day 60 and observed that loss of Notch3 induced a significant reduction of tumor-free survival (Fig. 3B). MMTV-Neu tumors were composed of well delimited, infiltrating, non-capsulated, highly cellular, multilobular tumors, and no evident modification could be seen in their histopathology in the absence of Notch3 (Fig. 3C). Next, we assessed by immunohistochemistry different markers of angiogenesis, proliferation, cell death and immune infiltrate. We saw no modification of CD31, CD8, cleaved-Caspase-3 staining between MMTV-Neu/Notch3^+/+^ and MMTV-Neu/Notch3^lacZ/lacZ^ phenotypes (Supplementary Fig. S3). Interestingly, the latter displayed a significant increase in Ki67 (Fig. 3D) showing that Notch3 null tumors were more proliferative.

### Notch3 loss induces an increase in proliferation in mammary gland tumors via Mybl2 regulation

At the molecular level, we further performed RNA-sequencing on murine tumors to verify whether loss of Notch3 was associated with a known Notch gene signature (Supplementary Table S2). We first assessed the Notch hallmark signature that was not significantly downregulated in Notch3 null tumors (Supplementary Fig. S4A). We then focused on a previously published Notch3-related signature [12] compiled using genes downregulated by Notch3-specific antagonist antibodies, and found that this signature was negatively enriched in Notch3-null tumors. We next tested a Notch3-independent DAPT-regulated signature, which was not lost in Notch3 null tumors, showing that Notch3 contributes to a specific signature in this context. The Notch signature published by Boelens et al. [25] was also more strongly expressed in Notch3-positive tumors.

**Fig. 4:**
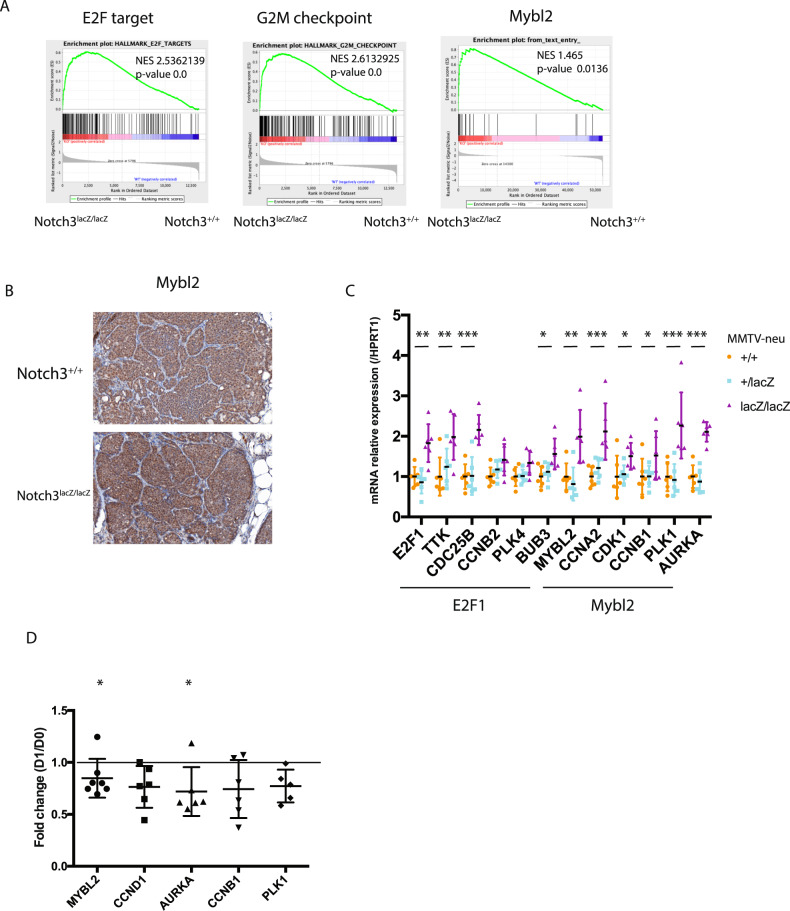
Notch3 is regulating a Mybl2 signature in mammary gland tumors. **A** GSEA analyses of E2F target, G2/M Checkpoint and Mybl2 signature comparing Notch3 wt and Notch3 null (Notch3lacZ/lacZ) tumors in MMTV-Neu induced tumors. **B** Immunohistochemistry for Mybl2 protein in Notch3 wild type and null tumors. **C** mRNA expression of E2F1 and Mybl2 targets in tumors dissected from MMTV-NEU; Notch3^+/+^ (+/+), MMTV-NEU; Notch3^lacZ/+^ (+/–) and MMTV-NEU; Notch3lacZ/lacZ tumors (–/–). **D** mRNA q-RT PCR analysis of Mybl2 and target genes in organoïds expressing N3ICD upon doxycyclin treatment (D1). Expression is expressed as fold change between N3ICD expressing and control organoïds of mRNA expression standardize to HPRT1.

We next assessed if Notch3 affected the cell-of-origin signature of tumors. As previously published, MMTV-Neu tumors showed a luminal progenitor cell signature that was not affected by loss of Notch3 (Supplementary Fig. S4B). Further analysis by single-sample GSEA, identified that Notch3 null tumors expressed a basal-like signature with a significantly decreased luminal A signature (Supplementary Fig. S4C). We were then interested in the effects of Notch3 on genes from the PAM50 gene signature, which constitutes the basis of breast tumor molecular classifications, and observed that this signature was able to segregate tumors according to Notch3 status (Supplementary Fig. S4D). We also observed a significant enrichment in Mybl2, Ube2c, and Rrm2, genes belonging to the basal-like cluster, in Notch3 null tumors, which was confirmed by quantitative RT-PCR (Supplementary Fig. S4E). Confirming observations by Lafkas et al. [19] we also observed a downregulation of CDKN1A and CDKN2A in Notch3 null tumors (Supplementary Fig. S4F). GSEA also highlighted an enrichment in cell cycle regulators E2F1, G2M checkpoints and Mybl2 signature in Notch3 null tumors (Fig. 4A). Immunohistochemical staining of these tumors then revealed an upregulation of Mybl2 expression and enhanced proliferation (MKI67 staining) (Fig. 4B and Supplementary Fig. S5). Quantitative RT-PCR further validated the increase in E2F1 and Mybl2 target genes in Notch3-deficient tumors (Fig. 4C). To further determine whether Notch3 expression could directly affect Mybl2 and the expression of its target genes, we investigated the effect of Notch3 expression in MDA-MB-231 cells engineered to express Notch3 upon doxycycline expression (Fig. 3A). Induction of Notch3 in organoids displayed a decrease in Mybl2 expression and that of its target genes (Fig. 4D). These experiments show that activation of Notch3 leads to decrease proliferation via Mybl2 reduction.

### Notch3 controls a HeyL-Mybl2 axis limiting tumor cell proliferation

**Fig. 5:**
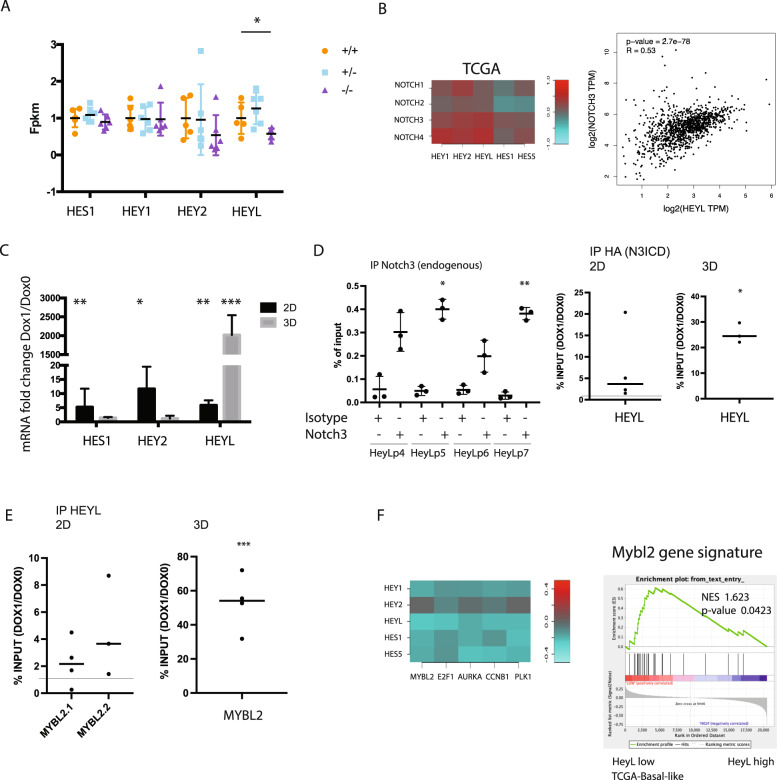
Notch3 is regulating Mybl2 via HeyL-mediated inhibition. **A** mRNA expression of Notch target genes in tumors from MMTV-NEU; Notch3^+/+^ (+/+), MMTV-Neu/Notch3^lacZ/+^ (+/–) and MMTVNEU/Notch3^lacZ/lacZ^ tumors (–/–) (*n* = 6 mice in each genotype). **B** Pearson coefficient of correlation between expression of Notch receptors and Notch traget genes in the TCGA-BRCA dataset and correlation between Notch3 and HeyL. **C** Expression of HES1, HEY2 and HEYL following N3ICD induction in MDA-MB231 cultured in 2D or in organoïds (3D) (*n* = 3 in each conditions). **D** PCR for HEYL promoter using 4 different sets of primers were realized in chromatin immunoprecipitated with Notch3 antibody in MCF7 cells (*n* = 3 for 4 pairs of primers) and with HA antibody in MDA-MB231 cells treated with Doxycyclin (DOX1) to induce N3ICD compared with non induced cells (DOX0) in 2D (*n* = 4 with one pair of primers and in 3D (one experiment with 3 different pairs of primers). **E** PCR for MYBL2 promoter after immunopreicipitaion of HeyL using two different set of primers in 2D (*n* = 4 for each pairs of primers) and in 3D with 4 different pairs of primers in one experiment. **F** Pearson coefficient of correlation between Notch target genes and Mybl2 in the TCGA dataset and GSEA for the Mybl2 signature comparing the HeyL high and HeyL low basal-like tumors in the TCGA-BRCA dataset.

To further characterize the Notch3 signaling and its effects on cell proliferation, we sought Notch3 target genes that could be involved in Mybl2 downregulation. Among well-known Notch target genes, only Hey2 and HeyL were downregulated in Notch3 null tumors, HeyL being the only significant downregulated (Fig. 5A). Furthermore, in the TCGA-BRCA dataset, Notch3 was more strongly correlated to HeyL than to other Notch target genes (including Hey1, Hey2, Hes1 and Hes5) (Fig. 5B). To verify the relationship between Notch3 and HeyL in vitro, we enforced N3ICD expression in MDA-MB-231 cells cultured in 2D and in 3D. Interestingly, although Hes1, Hey2 and HeyL expression reached similar levels in 2D culture condition, the expression of HeyL alone drastically increased in 3D organoid culture (Fig. 5C). This finding raises awareness to the differential regulation of Notch signaling in 2D versus 3D culture and the importance of culture conditions when studying juxtacrine pathways. To confirm the regulation of HeyL by Notch3 we performed chromatin immunoprecipitation with Notch3 antibody in MC7 cells, which express constitutive Notch3. We confirmed direct binding of endogenous Notch3 on HeyL promoter with 4 different pairs of primers (Fig. 5D). We next confirmed binding of N3ICD on HEYL promoter in MDA-MB231 cells when N3ICD was induced following DOX treatment both in 2D and in 3D. Interestingly enrichment of Notch3 on HEYL promoter was stronger in 3D. This show that HeyL is a direct target of Notch3 in breast cancer cell lines. We next focus on the regulation of Mybl2 by HeyL. We therefore looked for Chip-seq data published with HeyL and found that in HEK293T cells, Heisig et al., observed a significant peak in Mybl2 promoter [26]. We next looked by chromatin immunoprecipitation upon N3ICD expression in MDA-MB231 cells. We could show that HeyL directly binds to the promoter of Mybl2 both in 2D and in 3D (Fig. 5E). Importantly, the binding of HeyL on Mybl2 promoter was stronger in 3D.

We finally sought the correlation between HeyL and Mybl2 in the TCGA database. Among Notch target genes, HeyL was the most inversely correlated to Mybl2 and to E2F1 (Fig. 5F). Furthermore, GSEA comparing HeyL low and HeyL high patients confirmed enrichment of Mybl2 in HeyL low patients (Fig. 5F). Of note, we also observed a correlation in the expression of Notch3 and HeyL in single-cell data from normal mice mammary gland but only in luminal cells and not in stromal cells (Supplementary Fig. S6). This expression of Notch3 and HeyL is further anti-correlated with Mybl2, which is mainly expressed in basal cells (Supplementary Fig. S6). Altogether, these data, show that Notch3, in breast cancer cells, induces a strong expression of HeyL that leads to downregulation of Mybl2, which leads to decreased proliferation.

## Discussion

The role of Notch signaling in cancer has been extensively studied, with both oncogenic [27] and tumor suppressive [28] features. This dual role of Notch depends on its function in the cell of origin [29]. In the mammary gland, Notch3 marks luminal progenitors and limits their proliferation [19]. By using the single-cell data from the mouse atlas, we observed Notch3 expression in luminal cells (Supplementary Fig. S5), which was supported by beta-galactosidase staining of mammary glands of Notch3^LacZ/+^ mice (data not shown). With immunohistochemistry of human breast tissue, we observed a strong, sparse staining of basal myoepithelial cells and a weaker staining of luminal cells. This apparent discrepancy with what is observed in the mouse mammary gland could be explained by the fact that the low proportion of basal cell expressing Notch3 is not visible with single-cell sequencing. In human tumor single-cell analysis, strong Notch3 expression is maintained in the stromal compartment. However, in contrast to the role that has been described for stromal Notch3 in lung cancer, we observed here that stromal Notch3 had no impact on patient’s survival (Fig. 1E). In contrast, expression of Notch3 by cancer cells had an impact and was of good prognosis (Fig. 1E). The mixed expression and contribution of Notch3 in stromal or cancer cells points to the importance to study survival impact with protein staining rather than RNA-seq bulk sequencing. This loss of Notch3 in epithelial cells during transformation may be associated with a tumor suppressive role of Notch3. In favor of this role, we observed that loss of Notch3 induced an acceleration of tumor initiation (Fig. 3C). We also propose a mechanism for Notch3 loss in breast cancers, by demonstrating that Notch3 promoter’s methylation is negatively correlated to its expression (Fig. 2 and supplementary Fig. 2). This observation is confirmed by other study and Notch3 loss in breast cancers may thus be associated with epigenetic remodeling of tumors. Epigenetic modifications are also associated with mammary gland stem cells differentiation and Notch3’s expression is modulated by epigenetic factors [30, 31].

Notch3 null tumor showed increased proliferation as assessed by immunohistochemistry and RNA-seq signature analysis (Figs. 3E and 4). As described in Lafkas et al. [19] we observed that loss of Notch3 led to a decrease in p21 (Cdkn1) and Arf (Cdkn2a) potentially accelerating proliferation (Supplementary Fig. S3F). Regulation of p21 by Notch3 has also been proposed to act as a tumor suppressor in breast cancer by inducing senescence [14]. Although we saw a regulation of p21 by Notch3 both in tumors and in vitro in cells over-expressing Notch3, no evidence in the literature point to a role of senescence in the initiation step of tumorigenesis in the MMTV-Neu mice model but this would require further investigations as the increased proliferation of cancer cells observed in Notch3-null tumor may be associated with an early bypass of senescence. We focused here on the effect of Notch3 on Mybl2 expression. Indeed Mybl2 is an important regulator of cell cycle and has an important role in breast cancer [32, 33]. We showed that Mybl2 was upregulated in Notch3 null tumors and demonstrated that among Notch3 target genes, HeyL was lost in these tumors and associated to Mybl2 signature in TCGA-BRCA basal-like tumors. We further showed that Mybl2 promoter was bound by HeyL upon N3ICD expression. Interestingly, Notch-regulated genes were different when assessing genes in 2 or 3D pointing to the importance of culture set-up when assessing oncogenic features. Given the role of forces in Notch signaling regulation [3], it is likely that Notch signaling will be affected by matrices and culture conditions. We also observed in our RNA-seq data that a Notch3-specific signature was significantly altered in Notch3 null tumors (Supplementary Fig. 3), suggesting that Notch3 loss, at least on established tumors might be due to affecting Notch signaling. However, broader signatures established by DAPT treatment or the Notch Hallmark signature were not affected in Notch3 knocked-out tumors (Supplementary Fig. 3), showing that the absence of Notch3 is not sufficient to restrain the canonical Notch signaling mediated by the other Notch receptors.

Altogether, we provide evidence for a new target of Notch3, which have a major importance in regulating proliferation and tumor initiation. This regulatory pathway may be involved in other settings such as stem cell maintenance and differentiation in which Notch signaling is involved. In terms of cancer therapy, this study therefore points to the fact that Notch signaling role may be different in tumor initiation and progression and reinforce the need for patient stratification for targeted therapies aiming at inhibiting Notch signaling.

## Material and methods

### Mice

Experiments with mice were conducted in the AniCan Animal Facility of the Cancer Research Center of Lyon (CRCL) in agreement with the local ethics comity (CECCAPP, Comité d’Evaluation Commun au PBES, à AniCan, au laboratoire P4, à l’animalerie de transit de l’ENS, à l’animalerie de l’IGFL, au PRECI, à l’animalerie du Cours Albert Thomas, au CARRTEL INRA Thonon-les-Bains et à l’animalerie de transit de l’IBCP). C57BL/6 LacZ Notch3 knock-in mice were generously given by Silvia Fre and Spyros Artavanis-Tsakonas and have been previously characterized [24]. Back-crossing of these mice with Balb/c mice was done in order to obtain a Balb/C pure genetic background. Balb/c LacZ Notch3 mice were crossed with Balb/MMTV-Neu mice [23]. Mice were monitored for tumor development every two days. Once tumors were palpable, mice were reared for 30 days, sacrificed and tumors dissected. Mice were sacrificed before the end of the experiment if necessary, according to animal care guidelines. No animals were excluded from the study. No randomization was performed. Breeding was carried out in order to obtain the same amount of controls and KO animals in littermates. Investigator was blinded while assessing tumor occurrence in mice mammary gland since genotype was carried out by another investigator.

### Immunohistochemistry analysis

Immunohistochemistry was performed on 4-μm-thick sections of formalin-fixed, paraffin-embedded and heat-treated (for antigen retrieval) tissues (DakoCytomation). Sections were stained with hematoxylin-eosin-safran and with anti-Notch3 (Cell signaling, D11B8) anti-CD31 (anaspec (53332)), anti-Ki67 (eurobio, ref M3064). Positive cells were counted on whole sections using HALO software. Investigators were blinded when analyzing immunohistochemistry staining with Halo software.

### Cell culture

All cell lines were obtained from the ATCC. The human MDA-MB231 cell line is derived from the pleural effusion of a breast cancer metastatic site. MDA-MB231 cells were stably transfected using the FuGENE HD reagent (Promega), according to the manufacturer’s instruction with the Tet-pITR-puro-GFP plasmid, either empty and served as a control or containing full length Notch3-WT. Notch3 was tagged with HA. Cell lines were maintained in Dulbecco’s modified Eagle’s medium (DMEM media) complemented or not with 10% heat inactivated fetal bovine serum (FBS), 1% penicillin/streptomycin (P/S) and 4% gentamycin. Cells were cultured at 37 °C and 5% CO_2_. Notch3 expression was induced by Doxycycline (Dox) at 0.25 µg/mL or 1 μg/ml.

### Western blot

Cell pellets were harvested by centrifugation at 4 °C at 1400 × *g* during 5 min.

Cells were lysed in SDS buffer (2% SDS, 150 mM NaCl, 50 mM Tris-HCl, pH 7.4), then sonicated and centrifuged at room temperature for 5 min at 10,000 × *g*. The supernatant containing the extracted proteins was retained. Protein concentration was measured with the BCA assay kit (Pierce Biotechnology, Rockford, IL, USA) using bovine serum albumin (BSA) as a standard according to the manufacturer’s instructions. The same quantity of protein was loaded onto Biorad precast gels, transferred to a nitrocellulose membrane and blocked for 1 h in 5% milk. The following primary antibodies were used: anti-Notch3 (1:1000 dilution, Cell Signaling #5276 (D11B8)), anti-HA (1:5000 dilution, Sigma H4908) and anti-actin (1:5000 dilution, Sigma A3854).

### Soft-agar assay

A single-cell suspension of 30,000 MDA-MB231-pitr1-Notch3 cells selected for their high expression of GFP by flow cytometry in 1.5 mL 0.45% agarose (SeaPlaqurAgarose Lonza, 50100, lot 0000287875) was seeded onto 6-well plates containing a 0.9% agarose base. Doxycycline at 0.25 or 2 µg/mL was added to the cell suspension and at every medium change. Medium was thereafter changed every 3–4 days. After 8 weeks of incubation, the medium was removed and following a PBS wash, the colonies were fixed in 4% PFA and 0.005% crystal blue for 1 h. Samples were washed 3 times in PBS for 10 min and visualized by microscopy. Images were acquired with a Zeiss Axio microscope and colonies were counted. Three independent experiments were analyzed.

### Organoïd culture

We conducted a 3D embedded assay using EHS geltrex as a cell support. Six-well-plates were coated with pre-chilled EHS for 30 min at 37 °C. For plating, 3.4 × 10^5^ MDAMB-231 cells inducible for N3ICD were resuspended in 2 mL of medium and plated on the coated surface for 30 min at 37 °C. Then, 2 mL of medium was mixed with 200 μL of EHS and added to cells in culture. 48 h later, some cells were treated with 1 μg/mL of doxycycline and cells were then maintained in culture for 8 days. Medium containing 1 μg/mL of doxycycline was changed every 2 days thereafter. To extract the 3D structures, we first washed each well with ice-cold PBS. Then, EHS was scraped with PBS-EDTA (10 mM) and cells were transferred to a conical tube and incubated at 4 °C on a shaker for 45 min. Cells were centrifuged at 115 × *g* for 2 min to pellet them and extract the RNA using NucleoSpin RNA extraction kit (Machery-Nagel).

### Chromatin immunoprecipitation (ChIP)

In all, 40 × 10^6^ MDAMB-231 cells were seeded 24 h before treatment or not with 1 μg/mL of doxycycline to induce the intracellular domain of Notch3 (N3ICD). Cells were scraped, centrifuged at 800 × *g* for 5 min and washed with 20 mL of cold PBS before fixation in 20 mL of paraformaldehyde (PFA) for 10 min at 4 °C. Cells were washed three times with cold PBS, resuspended in 9 mL of L1 buffer (50 mM Tris pH 8.0, 2 mM EDTA, 0.1% NP40, 10% Glycerol) for 5 min on ice. Cells were centrifuged at 2000 × *g* at 4 °C for 5 min and the pellet resuspended in 2 mL of L2 buffer (50 mM Tris pH 8, 5 mM EDTA, 1% SDS). The chromatin fragmentation was done by sonication at 20%, for 6 min with 1 s between each pulse then the debris were removed by centrifugation at 9400 × *g* for 5 min. DNA concentration was assessed by collecting 150 μL of fragmented DNA and mixing it with 2 μL of proteinase (10 mg/mL) and 6 μL of NacL (5 mM) for 30 min at 37 °C. Then, 2 μL of proteinase K (20 mg/mL) was added and incubated at 65 °C for 2 h. DNA was purified with NucleoSpin Gel and PCR clean-up (MN 740609.5), and 10 μg of DNA was diluted in 9 volumes of dilution buffer (20 mM Tris pH 8.0, 2 mM EDTA, 0.1% SDS, 1% NP40, 500 mM Nacl). 100 μL served as input. A pre-clear was conducted by adding of 30 μL of chip-Grade Protein G Magnetic Beads (Cell signaling) for 3 h at 4 °C. The supernatant was collected and 2 μg of antibody HeyL or HA (sigma-aldrich) was added and incubated overnight at 4 °C. To collect the immunocomplexes, 30 μL of beads were supplemented to the mix DNA/antibody and incubated for 30 min at 4 °C then centrifuge at 3400 × *g* for 1 min. Immunocomplexes were washed three times for 5 min with washing buffer (20 mM Tris pH 8.0, 2 mM EDTA, 0.1% SDS, 1% NP40, 500 mM NaCl) and three times with 1x TE buffer. The Ag/Ab complexes were extracted in 100 μL of elution buffer (1x TE buffer, 2% SDS) and reversion of crosslinking was done with proteinase A, NaCl and proteinase K then DNA purified with Nucleospin Gel and PCR clean-up. A q-PCR was then performed on input and Chip using the following primers:

MYBL2.1prom-F: AGGAGAGGAAGCAGGGAGAG

MYBL2.1prom-R: CATAGCGAAGACCGAGGAAG

MYBL2.2prom-F: TTTTGTCTCCCGCCTAATTG

MYBL2.2prom-R: CCGGAATGTTAAGGAGCAAA

HEYLprom-F: GCTCTCATGCAGCTTCCTTT

HEYLprom-R: GGCAACCCATCAAACTGTTC

HEYLprom4F: CATTACTGCATCTTCCCCGC

HEYLprom4R: AGACGTTGGCTCTGAGTTGA

HEYLprom5F: ACATACCCCAACTCTGCTCC

HEYLprom5R: TTGGCTCGCAACAAATCCAA

HEYLprom6F: CAAGACCCCACTGTGATCCT

HEYLprom6R: GCTGGGGTTGTTGTGTCTTT

HEYLprom7F: TAGTCAGTGAGAGGGTGGGT

HEYLprom7R: ACATAGTGTCTGCCTCGCTT

### Cut and run

200,000 of MDAMB-231 cells were seeded on 1.6 mL of Geltrex for 24 h then treated with 1ug/mL of doxycycline for 24 h. After treatment, 3D structures were extracted with PBS-EDTA (10 mM), filtered with a cell strainer 40 µM and spin at 300 × *g* for 3 min. Cells were resuspended in 1x Wash Buffer (10x Wash Buffer + 100x Spermidine + 200x Protease Inhibitor Cocktail) at room temperature then centrifuge for 3 min at 600 × *g*. These steps were repeated two times and the pellet was resuspended in 100 µL of 1x Wash Buffer. In parallel, 10 µL of Concanavalin A Magnetic beads were prepared by the addition of 100 µL of Concanavalin A bead Activation Buffer and placed on a magnetic rack for 2 min. These steps were repeated 2 times. Then, 10 µL of activated beads were added to the 100 µL sample and incubated for 5 min at room temperature. Samples were centrifuge at 100 × *g* for 2 s and placed on a magnetic rack to remove the liquid. 100 µL of Antibody Binding Buffer (100x Spermidine, 200x Protease inhibitor Cocktail, Digitonin Solution and Antibody Binding Buffer) and 2 µg of antibody was added to the complex overnight at 4 °C. The day after, samples were centrifuge at 100 × *g* for 2 s, placed on a magnetic rack for 2 min to remove the liquid and the complexes were resuspended in 1 mL of Digitonin Buffer (100x Spermidine, 200x Protease Inhibitor Cocktail, Digitonin Solution), these steps were repeated two times. 50 µL of pAG-MNase was added in each sample for 1 h at 4 °C. The samples were briefly centrifuge at 100 × *g* for 2 s and placed on a magnetic rack for 2 min to remove the liquid. The complexes were washed with 1 mL of Digitonin Buffer, placed on a magnetic rack to remove the liquid, and these steps were repeated two times. 150 µL of Digitonin Buffer was added to each complex and incubated on ice for 5 min. We activated the pAG-MNase by adding 3 µL of Calcium Chloride and incubated at 4 °C for 30 min. Then, we added 150 µL of 1x Stop Buffer (Digitonin Solution, RNAse A and 4x Stop Buffer) and incubated at 37 °C for 10 min. Samples were centrifuged at 4 °C for 2 min at 16,000 × *g* and placed on a magnetic rack for 2 min. The supernatant containing the enriched chromatin samples was transferred in a new tube. In parallel, Input samples were prepared by adding 200 µL of DNA extraction Buffer (Proteinase K, RNAse A) to the 100 µL input sample and incubated at 55 °C for 1 h with shaking. Then, samples were cooled on ice for 5 min and cells were lysed and chromatin fragmented by sonicating 1 min at 20%. The lysate was then clarified by centrifugation at 18,500 × *g* for 10 min at 4 °C and the supernatant was transferred in a new tube. DNA was purified with NucleoSpin Gel and PCR clean-up (MN 740609.5) and eluted in 20 µL of Nuclease free water.

### Bisulfite assay

DNA extraction from cells was performed using the DNA extraction kit (Machery-Nagel) according to the manufacturer’s instruction. The bisulfite reaction was done with the EpiTect Bisulfite kit (Qiagen) according to the manufacturer’s instruction. A PCR was done in a highly enriched region of the Notch3 promoter and further sequenced. The sequences were analyzed using the FinchTV Software to visualize the nucleotide peaks. The methylated cytosine residues were determined following sequence alignment.

### Real-time quantitative polymerase chain reaction (RT-qPCR)

mRNA extraction was performed using the NucleoSpin RNA kit (Machery-Nagel) according to the manufacturer’s instruction. cDNAs were generated with the iScript cDNA Synthesis kit (Biorad) according to the manufacturer’s instruction. Real-time quantitative polymerase chain reaction (RT-qPCR) was performed with FastStart TaqMan Probe Master Mix (Roche Applied Science) on LightCycler 480 machine (Roche Applied Science).

### RNA sequencing (RNAseq) and GSEA analysis

mRNA samples extracted from six tumors of each genotype were sequenced by the CLB sequencing platform. Full Fastq files were aligned against the reference mouse genome using (grcm38). Gene set enrichment analysis was performed using GSEA software. Enriched GO gene sets were selected and specific signatures were selected from the literature (Supplementary Table 2) and the broad institute database.

### TCGA data analysis

TCGA datasets were downloaded, assembled and processed using TCGA-Assembler (data download in December 2014). RNAseqV2 data were used and RPKM values were imported for transcriptomic analysis. Illumina Infinium Human Methylation450 Bead Chip data was used for the measurement of methylation status of CpG sites. The data was processed using R statistical v 3.3.1 software (R Development Core Team, R: A language and environment for statistical computing. R Foundation for Statistical Computing, Vienna, http://www.R-project.org.). R scripts are available on request. Survival analysis were done using the Kaplan-Meier method using as end point either overall survival (OS) or the tumor-free survival (TMS).

### Experimental design and statistical analysis

Sample size was chosen considering at least three independent experiment for trivial in vitro technics with cell lines (RT-qPCR, western blot). For chromatin immunoprecipitation we performed independent experiment with at least two pairs of primers (up to four). For animal studies we studied as many littermates as possible for 1 years (*n* = 24; 28 and 12).

Statistical analysis was performed using Prism v 6.0 (GraphPad software Inc., San Diego, CA) or R statistical v 3.3.1 software. For analysis of different measurements, a normality test was conducted, when the number of samples was sufficient. Variance was assessed to test for possible statistical analysis. For samples following a normal Gaussian distribution, a Student *t*-test was applied, either paired or unpaired, depending on the experimental data. When samples did not pass the normality test, a non-parametric test was applied (Mann–Whitney for unpaired samples and Wilcox on signed-rank test for paired samples). The correlation between the expression and methylation of Notch3 was done using a Cox test. The statistics on IHC of normal tissues compared to tumor tissues of patients with respect to cell localizations was done using a Chi-squared test of conformity. Differences between groups of the survival analysis were tested by log-rank tests. *p < 0.05; **p < 0.01; ***p < 0.001.

## Supplementary information

Supplemental figures

Checklist

additional data

## Data Availability

All data generated or analyzed during this study are available from the corresponding author on reasonable request.
