# Supplementary material for: Notch3 regulates Mybl2 via HeyL to limit proliferation and tumor initiation in breast cancer
Source: Cell Death Dis. 2023 Feb 28;14(2):171. doi: 10.1038/s41419-023-05674-7 (PMC9975231; doi:10.1038/s41419-023-05674-7)
Supplement: Supplementary file 1 — Supplemental figures [file 41419_2023_5674_MOESM1_ESM.pdf]

Brahim et al., supplementary Fig 1

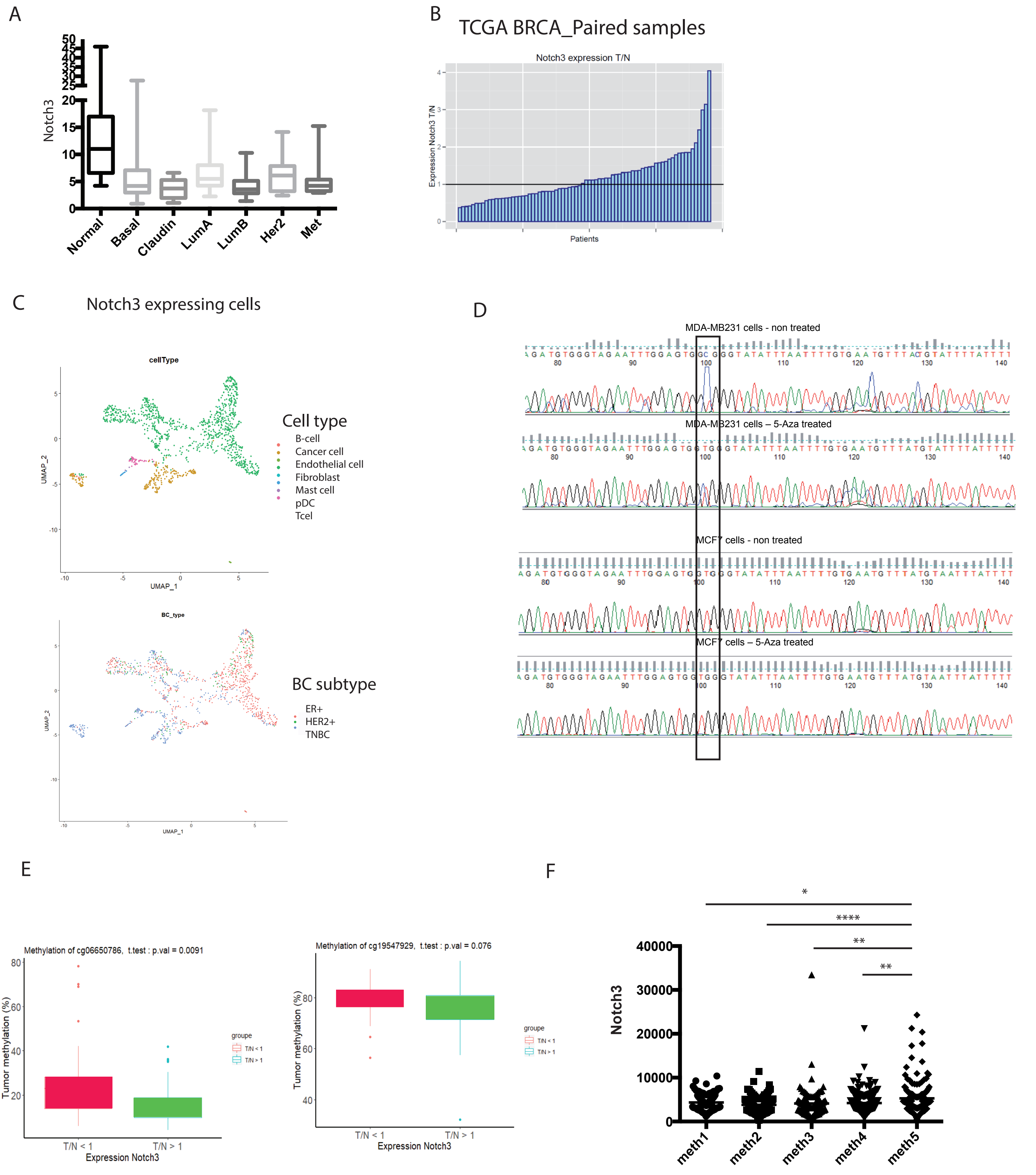

Supplementary Figure 1. Notch3 expression in Breast cancers.

A. Notch3 mRNA expression in the different subtypes of Breast cancers (GSE3165 dataset). B. Notch3 mRNA expression Tumor/Normal ratio in paired samples (TCGA dataset). C. Notch3-expressing cells in single-cell analysis of breast cancers from Bassez et al.,. D. Sequencing data of DNA treated with bisulfite of cells from MDA-MB231 and MCF7 cells treated or not with 5-aza. E. Methylation percentage of the indicated CpG probe of tumors of patients in which notch3 is decreased (T/N<1) or increased (T/N>1) in the tumor. F. Notch3 expression in patients sample from the TCGA belongling to the different methylation status of the tumor

Brahim et al., Supplementary figure 2

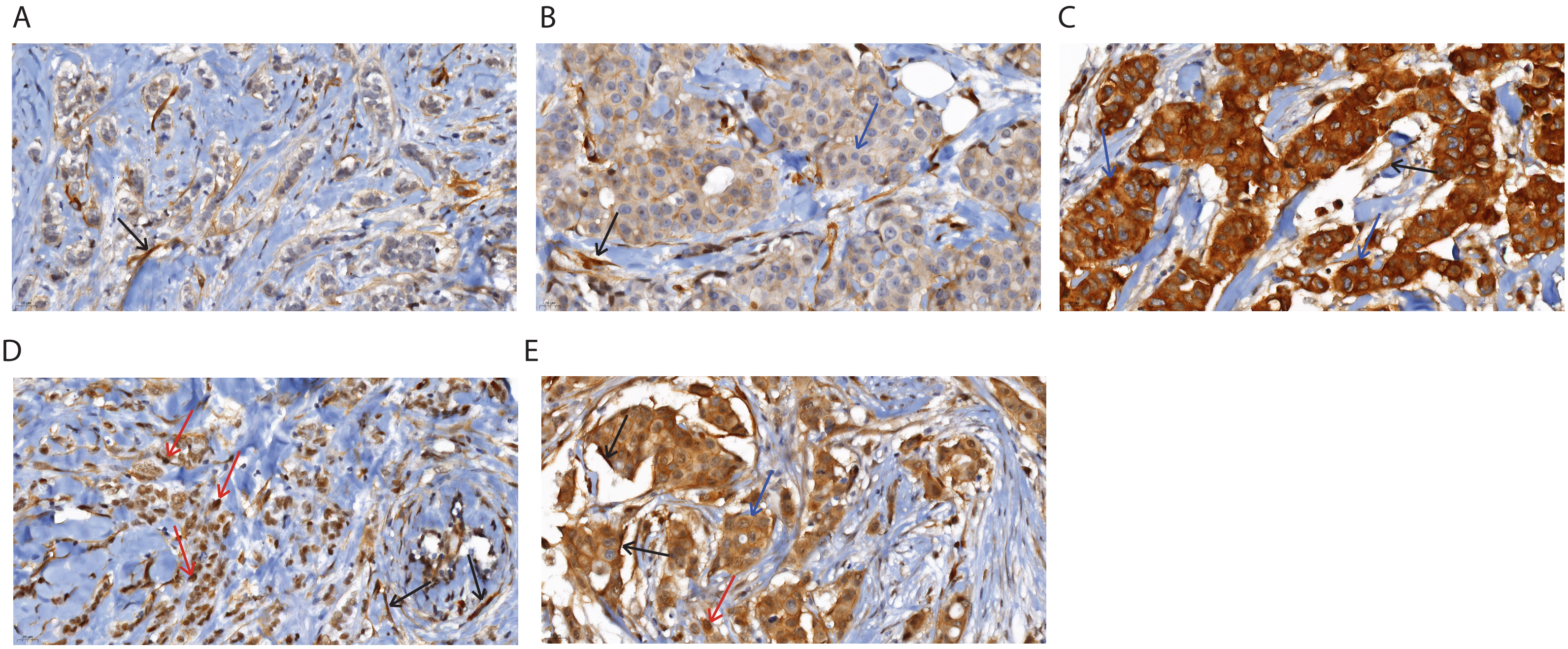

Supplementary figure 2. Different aspects of Notch3 staining in breast cancer patients samples. A. Sample showing no staining in cancer cells and a staining of medium intensity in myofibroblast (black arrow). B. Sample with a medium intensity in cytoplasm of cancer cells (blue arrow) and a strong intensity in myofibroblasts (black arrow). C. Sample with a strong staining in cytoplasm of cancer cell (blue arrow) and medium staining in myofibroblasts (black arrow). D. Sample with a high staining of nuclei of cancer cells (red arrow) and staining in myofibroblast (black arrow). E. Sample with staining in the cytoplasm (blue arrow) in all cancer cells and staining in the nuclei (red arrow) for 40% of cancer cells.

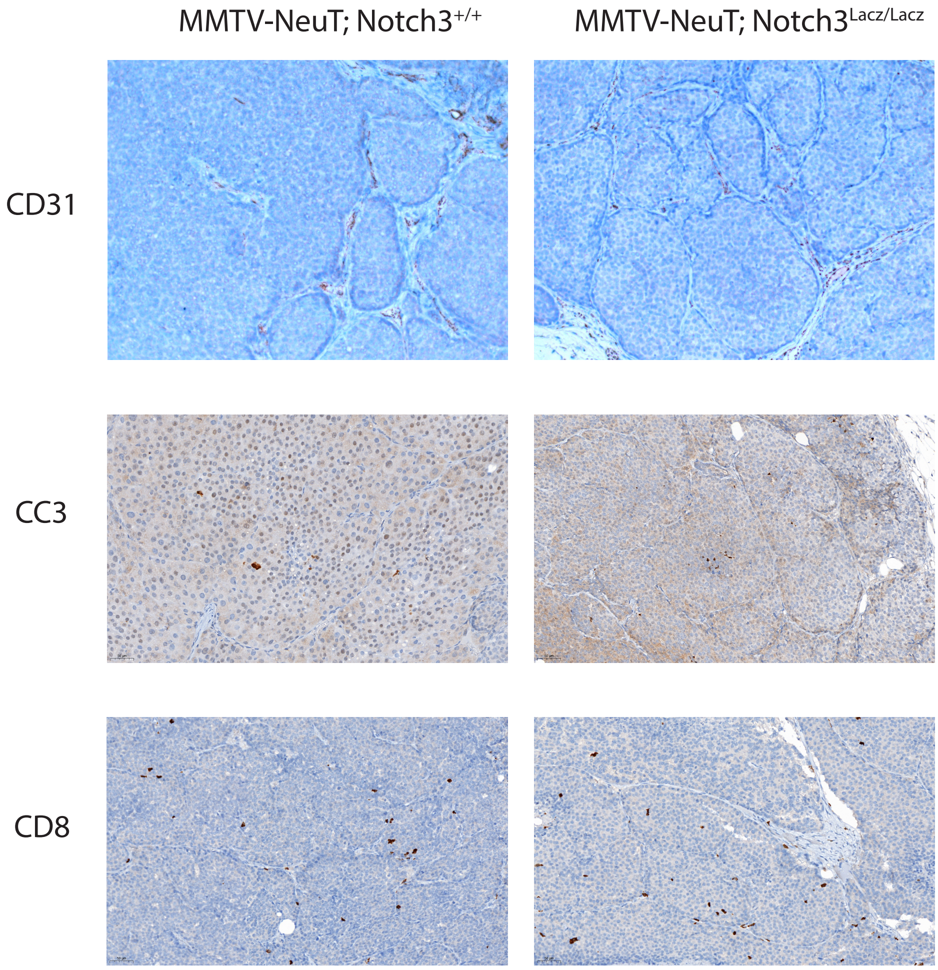

Supplementary figure 3. Immunohistochemichal analyis of CD31, cleaved caspase-3 (CC3) and CD8 expression in MMTV-Neu tumor with wild type Notch3 (Notch3$^{+/+}$) or with Notch3 loss of function (Notch3$^{lacZ/lacZ}$).

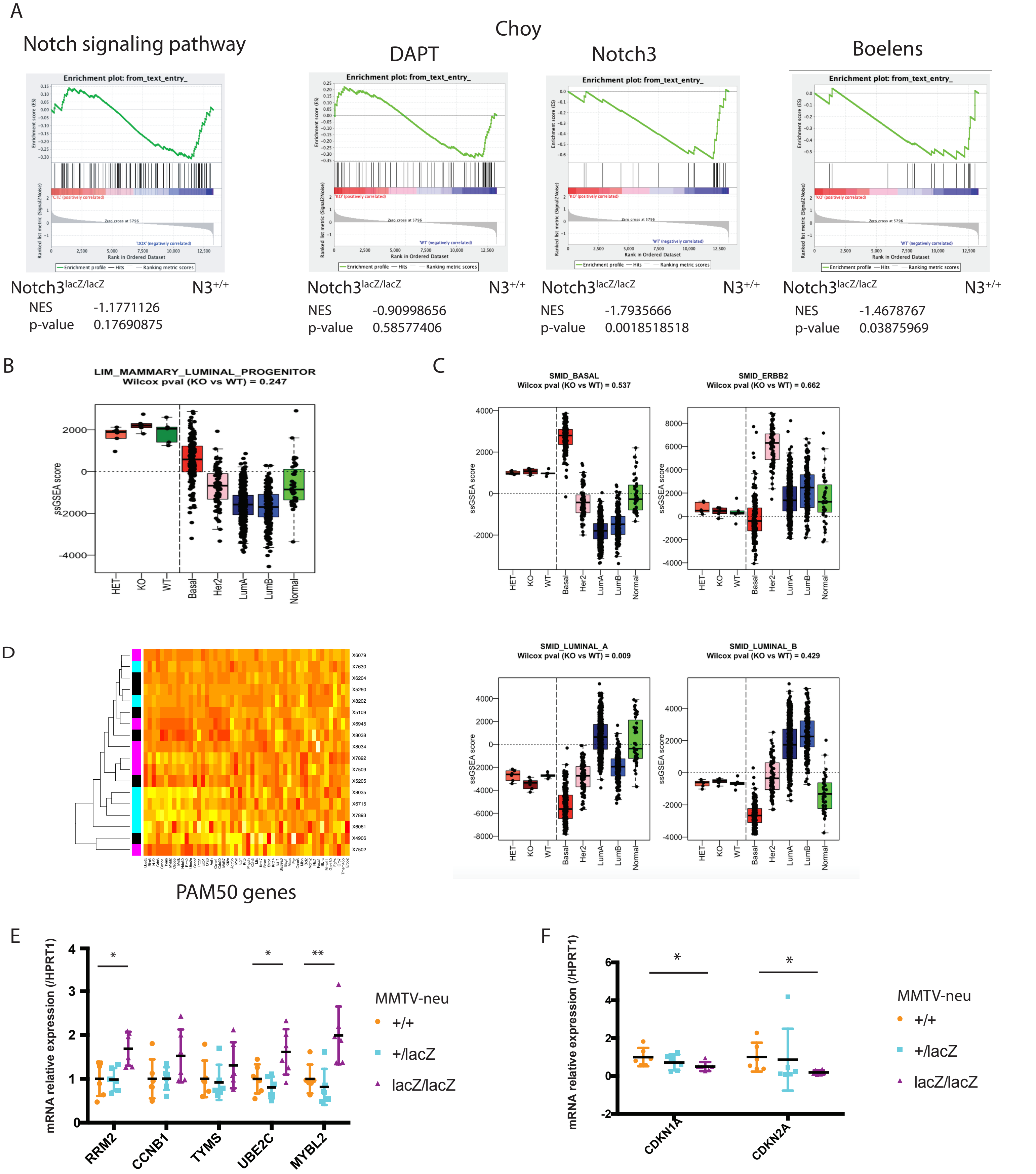

Supplementary Figure 4. RNAseq of breast tumors developed in MMTVNeu/Notch3$^{+/+}$, MMTVNeu/Notch3$^{+/LacZ}$ and MMTVNeu/Notch3$^{LacZ/LacZ}$ mice.

A. Gene Set Enrichment Analyses (GSEA) of different Notch signaling pathway gene set (Normalized Enriched score (NES) and p-value of the different Notch signatures indicated)), between Notch3 wild type and Notch3 null tumors. B, C, single-set gene set enrichment analysis (ssGSEA) of the indicated signatures comparing the MMTV-Neu tumors of the different genotypes (Notch3$^{+/lacZ}$ (HET); Notch3$^{lacZ/lacZ}$ (KO)) with the tumors from the TCGA BRCA database according to breast cancer subtype. D. Hierarchical clustering of the mammary glands tumors obtained in mice of the different indicated genotypes using the PAM50 genes. E, F. Q-PCR mRNA expression of indicated genes in tumors dissected from MMTV-NEU/Notch3$^{+/+}$, MMTV-NEU/Notch3$^{lacZ/+}$ and MMTV-NEU/Notch3$^{lacZ/lacZ}$ tumors.

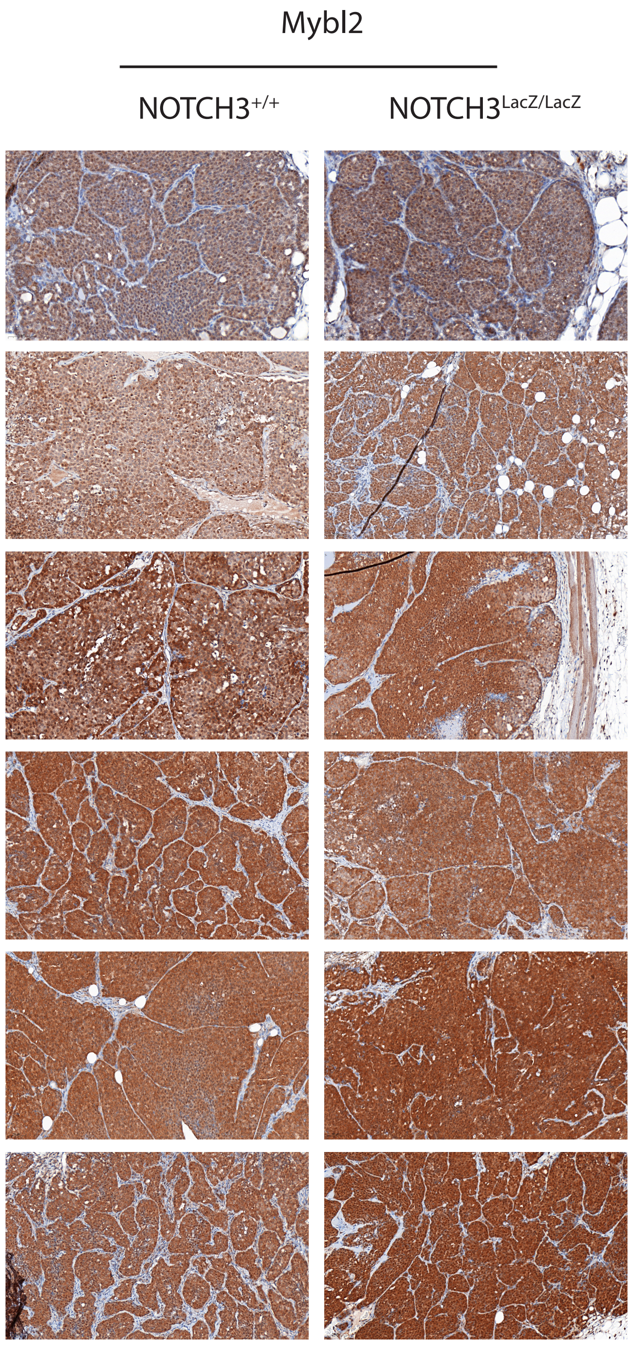

Supplementary figure 5. Immunohistochemichal analyis of Mybl2 expression in MMTV-Neu tumor with wild type Notch3 (Notch3$^{+/+}$ or with Notch3 loss of function (Notch3$^{lacZ/lacZ}$).

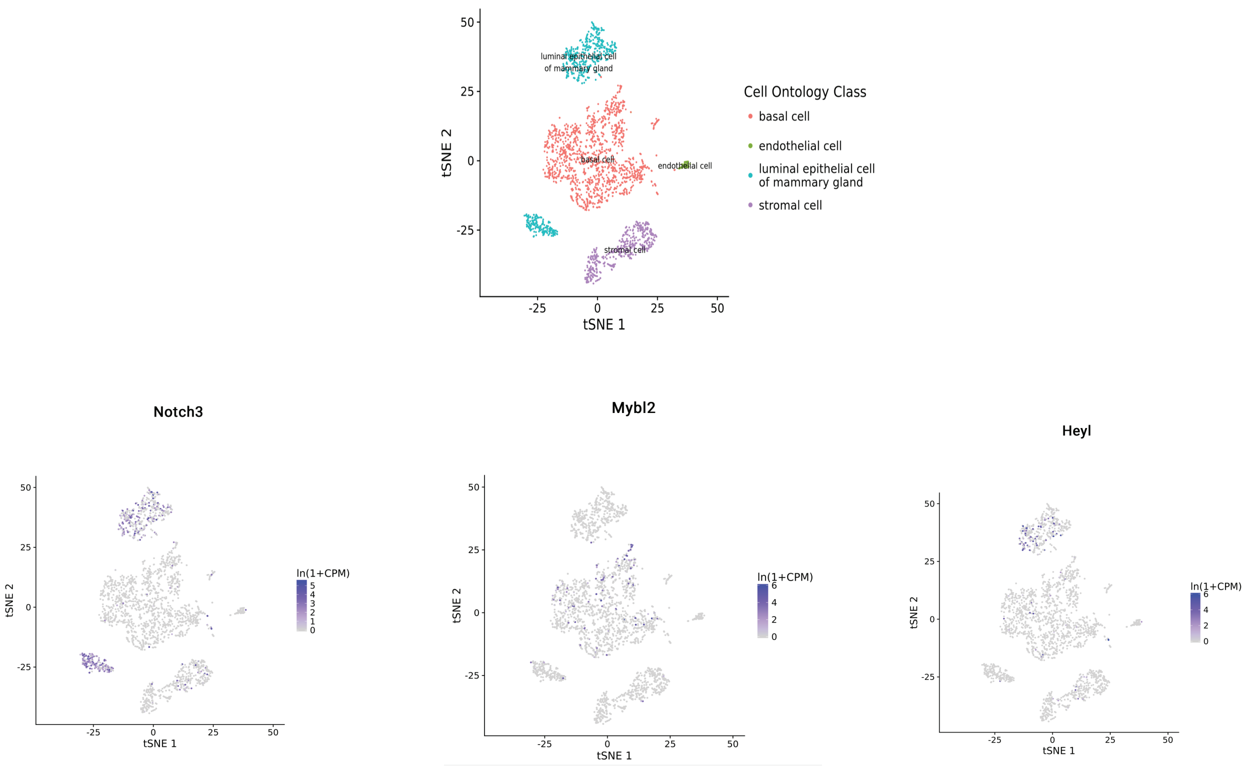

Supplementary figure 6. Notch3 is expressed in luminal cells, correlates with HeyL and is anti-correlated to Mybl2.
Single cell deata from mouse atlas showing Notch3, Mybl2 and HeyL expression in mice mammary gland

probes in CpG island	position of CpG island or cgxxxxxxxx probes on GRCh38	CpG position within the cgxxxxxxxx probe (GRCh38)	localisation *Notch3* gene	Relation to CpG Island	Pearson's correlation coefficient: methylation ~ expression	p value
**1 st CpG Island**	**chr19:15,199,709-15,201,216**		**promoter to intron 1**		**-0.24 ✢**	**4.72e-12**
cg19547929	chr19:15,201,362-15,201,483	chr19:15,201,422	promoter	S_Shore	**-0.18**	**5.23e-07**
cg06650786	chr19:15,201,258-15,201,379	chr19:15,201,318	promoter	S_Shore	**-0.26**	**7.27e-14**
cg24703175	chr19:15,200,717-15,200,838	chr19:15,200,777	intron 1	Island	**-0.1**	**0.004**
cg17498321	chr19:15,200,433-15,200,554	chr19:15,200,493	intron 1	Island	**-0.12**	**6.39e-04**
cg02584678	chr19:15,199,649-15,199,770	chr19:15,199,709	intron 1	Island	**-0.14**	**6.96e-05**
**2 nd CpG Island**	**chr19:15,195,433-15,196,300**		**intron 2**		**-0.25 ✢**	**1.41e-12**
cg20712669	chr19:15,197,631-15,197,752	chr19:15,197,691	intron 1	S_Shore	**-0.21**	**2.64e-09**
cg27320207	chr19:15,196,186-15,196,307	chr19:15,196,246	intron 2	Island	0.03	0.385
cg17075538	chr19:15,196,021-15,196,142	chr19:15,196,081	intron 2	Island	-0.05	0.151
cg13673837	chr19:15,195,799-15,195,920	chr19:15,195,859	intron 2	Island	-0.04	0.289
cg15075267	chr19:15,195,461-15,195,582	chr19:15,195,521	intron 2	Island	-0.03	0.346
cg08529654	chr19:15,195,067-15,195,188	chr19:15,195,127	intron 2	N_Shore	**-0.22**	**1.45e-10**
cg22311879	chr19:15,191,673-15,191,794	chr19:15,191,733	intron 5	N_Shelf	**-0.08**	**0.026**
**3 rd CpG Island**	**chr19:15,189,021-15,189,356**		**exon 7 to exon 8**		-0.07 ✢	0.059
cg01483459	chr19:15,189,295-15,189,416	chr19:15,189,355	exon 7	Island	**-0.11**	**0.002**
cg11358549	chr19:15,188,996-15,189,117	chr19:15,189,056	exon 8	Island	-0.04	0.262
cg01282080	chr19:15,187,207-15,187,328	chr19:15,187,267	exon 11	N_Shore	**-0.18**	**4.98e-07**
cg16048781	chr19:15,185,284-15,185,405	chr19:15,185,344	exon 14	N_Shelf	0.06	0.091
**4 th CpG Island**	**chr19:15,181,589-15,181,821**		**intron 16 to exon 17**		0.04 ✢	0.243
cg26880200	chr19:15,181,904-15,182,025	chr19:15,181,964	intron 16	S_Shore	**0.12**	**6.37e-04**
cg05682965	chr19:15,181,760-15,181,881	chr19:15,181,820	intron 16	Island	0.04	0.262
cg01643250	chr19:15,181,614-15,181,735	chr19:15,181,674	exon 17	Island	-0.01	0.701
cg15205568	chr19:15,181,745-15,181,866	chr19:15,181,805	intron 16	Island	0.01	0.679
**5 th CpG Island**	**chr19:15,177,534-15,178,100**		**intron 23 to exon 24**		**0.17 ✢**	**1.76e-06**
cg25535708	chr19:15,179,035-15,179,156	chr19:15,179,095	exon 22	S_Shore	-0.01	0.823
cg09265397	chr19:15,177,928-15,178,049	chr19:15,177,988	exon 24	Island	**0.08**	**0.023**
cg21514227	chr19:15,177,444-15,177,565	chr19:15,177,504	intron 24	N_Shore	**0.2**	**1.79e-08**
**6 th CpG Island**	**chr19:15,174,069-15,174,398**		**exon 25**		0.06 ✢	0.084
cg01814364	chr19:15,174,237-15,174,358	chr19:15,174,297	exon 25	Island	-0.05	0.12
cg19388017	chr19:15,174,229-15,174,350	chr19:15,174,289	exon 25	Island	0.04	0.228
cg14696996	chr19:15,174,210-15,174,331	chr19:15,174,270	exon 25	Island	-0.05	0.131
cg27191554	chr19:15,174,099-15,174,220	chr19:15,174,159	exon 25	Island	-0.01	0.832
cg22809798	chr19:15,173,982-15,174,103	chr19:15,174,042	intron 25	N_Shore	**0.12**	**7.61e-04**
**7 th CpG Island**	**chr19:15,170,342-15,170,756**		**exon 26 to exon 27**		-0.01 ✢	0.867
cg02904605	chr19:15,170,736-15,170,857	chr19:15,170,796	exon 26	S_Shore	-0.02	0.518
cg21726593	chr19:15,170,646-15,170,767	chr19:15,170,706	exon 26	Island	-0.002	0.961
cg02294312	chr19:15,170,486-15,170,607	chr19:15,170,546	exon 27	Island	**-0.1**	**0.003**
cg20688157	chr19:15,170,377-15,170,498	chr19:15,170,437	exon 27	Island	-0.06	0.116
cg09752223	chr19:15,170,329-15,170,450	chr19:15,170,389	exon 27	Island	0.03	0.466
cg08013143	chr19:15,167,269-15,167,329	chr19:15,167,329	exon 29	N_Shelf	**-0.11**	**0.002**
cg02272795	chr19:15,168,373-15,168,494	chr19:15,168,433	intron 28	N_Shore	**0.11**	**0.001**
cg18875435	chr19:15,160,139-15,160,260	chr19:15,160,199	exon 33		**0.14 ✢**	**1.11e-04**

✢ mean of probes methylation within the CpG Island per patient was correlated with Notch3 expression

Supplemental table 2

Notch signatures used in the study

Choy et al., Cancer Res. 2017 Mar 15;77(6):1439-1452.
DAPT
DNAJC6
TTF2
SLC4A4
GAS1
IL22RA2
GAL
RAB12
CCDC71L
TGM2
CDT1
MYBL1
CCDC58
NEIL3
WDHD1
UPP1
SERPINB7
RASSF10
ATAD2
C19orf33
ARL13B
ARHGAP11A
SLC16A1
TMEM156
WDR43
S100A3
TRIB2
EPB41L2
CLSPN
ID4
TAF4B
ID1
DACH1
EIF2S3
SLC16A6
TAF5
PPP3R1
UHRF1
ALDH1L2
SLC4A7
FAM43A
CPM
MCM10
MIS18A
TNC
TAF1A
DPP4
KRT5
GDA
FYN
NABP1
MYLK
THBS1
CAV1
ALPP
FAM216A
BCL11B
SOCS3
BRCA2
THOC6
COL15A1
CTH
UBA6
RAD54L
CLIP4
EVA1A
C20orf27
KLF9
JADE1
NCAM1
PLCL1

Choy et al., Cancer Res. 2017 Mar 15;77(6):1439-1452. NOTCH3
KRT6B
OLFML3
KCTD12
CGA
IER5
NA
PLD1
KRT6A
HS6ST2
JAG1
EPHB6
HES1
OLFM4
SLC7A5
SERPINB3
SLC45A3
SERPINA3
LIN9
MYC
ENOX1
GRIK2
STC2
SERPINB4
NRP1
GUCY1B3

Boelens et al., Cell. 2014 Oct 23;159(3):499-513
HES1
HEY2
CDKN1A
HES7
HEYL
HEY1
MFNG
DLL1
HES2
HES3
DLK1
JAG1
HES4
MAML1
HES5
DTX2
TNC

Broad institue Hallmark Notch signaling
APH1A
ARRB1
CCND1
CUL1
DLL1
DTX1
DTX2
DTX4
FBXW11
FZD1
FZD5
FZD7
HES1
HEYL
JAG1
KAT2A
LFNG
MAML2
NOTCH1
NOTCH2
PPARD
PRKCA
PSEN2
PSENEN
RBX1
SAP30
SKP1
ST3GAL6
TCF7L2
WNT2
WNT5A
