# Supplementary figures and images for: Notch3 regulates Mybl2 via HeyL to limit proliferation and tumor initiation in breast cancer
Source: Cell Death Dis. 2023 Feb 28;14(2):171. doi: 10.1038/s41419-023-05674-7 (PMC9975231; doi:10.1038/s41419-023-05674-7)

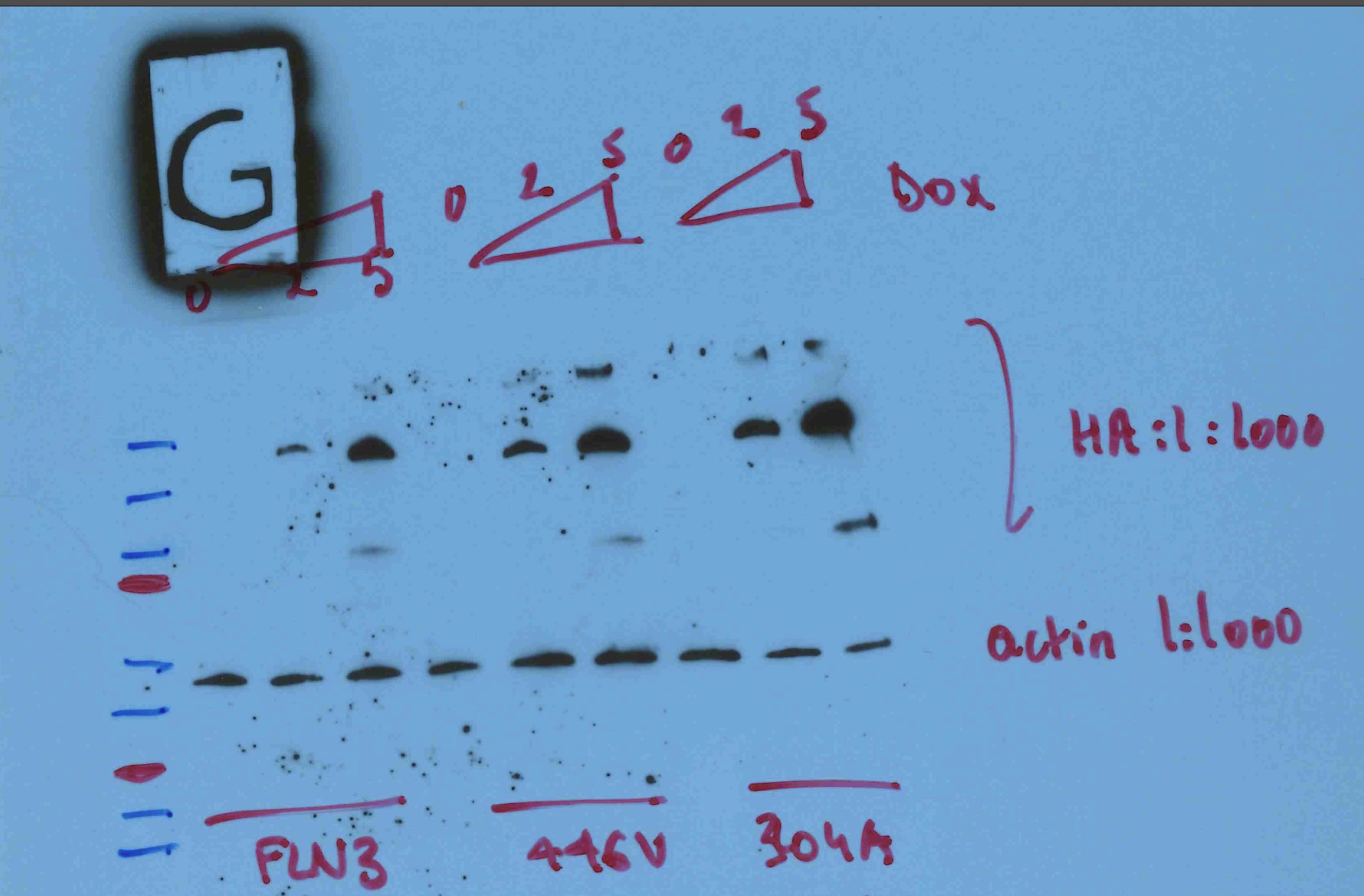

Supplement: Supplementary file 3 — additional data [file 41419_2023_5674_MOESM3_ESM.jpg]
